# A further step towards the practical application of quantum computing in chemistry

**DOI:** 10.1038/s42004-022-00727-y

**Published:** 2022-09-07

**Authors:** Libor Veis

**Affiliations:** grid.418095.1 0000 0001 1015 3316 J. Heyrovský Institute of Physical Chemistry, Academy of Sciences of the Czech Republic, v.v.i., Dolejškova 3, 18223 Prague 8, Prague, Czech Republic

**Keywords:** Quantum chemistry, Method development

## Abstract

Adiabatic state preparation (ASP) represents an efficient way of generating correlated wave functions on quantum computers for subsequent quantum simulation. Here, the author discusses recent work that numerically studied the performance of ASP on strongly correlated molecules and presented several approaches of improving the quality of prepared ground state wave functions.

Quantum computers undoubtedly represent one of the biggest technological promises of today. Despite complications inherently connected to storing and manipulating information in fragile quantum states of matter, quantum computing in principle offers enormous computational power, with the potential to exponentially speed-up computations of certain types of problems^1^. Finding the ground (and low-lying) electronic states of molecules, which is the fundamental task of quantum chemistry, is one such problem. Indeed, as mentioned by Takui et al.^2^, quantum computers have the potential to bring a paradigm shift in chemical research. It was R. Feynman, who realized that exact simulation of quantum systems (including molecules) on classical computers is hard and came up with the idea of a quantum computer^3^.

In fact, the basic equation of quantum chemistry—the electronic Schrödinger equation—in its exact form represents a many-body problem, whose computational complexity is well known to grow exponentially with the number of electrons. When searching for the exact *N*-electron wave function in a space of *M* molecular orbitals (*M* > *N*), one has to expand the wave function in a basis of all possible configurations (more precisely Slater determinants^4^), i.e., distribute *N* electrons in *M* molecular orbitals in all possible ways. The method which variationally optimizes wave functions of this form is called the full configuration interaction (FCI). Since the number of all possible configurations grows exponentially with the size of the problem (see Fig. 1), its cost is prohibitive and it is applicable only to the smallest molecules (e.g., diatomics).

**Fig. 1:**
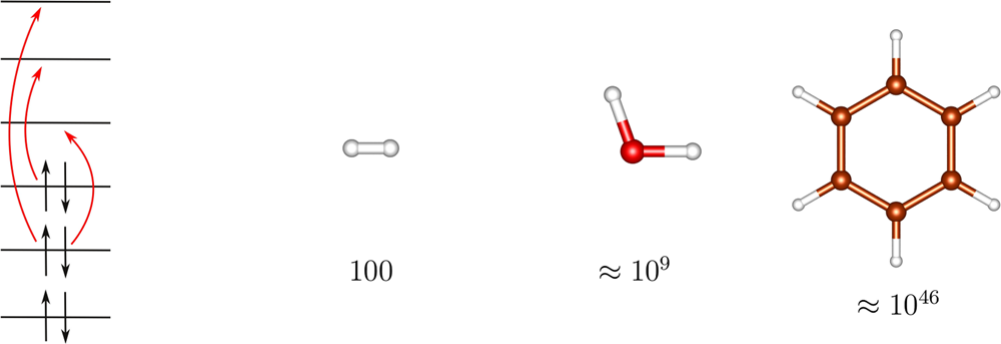
Exponential scaling of FCI. Scheme of formation of all possible configurations from the mean-field solution and exponential scaling of the resulting full configuration interaction (FCI) method demonstrated on the examples of H_2_, H_2_O, and C_6_H_6_ molecules. The numbers below the molecular structures represent the number of configurations (Slater determinants) in the FCI wave function expansions in double-*ζ* basis.

The basic unit of quantum information, a quantum bit (qubit), is a superposition state *c*_0_ | 0〉 + *c*_1_ | 1〉, with |0〉 and |1〉 being the abstract computational basis states and *c*_0_, *c*_1_ complex numbers satisfying the normalization. The state of a quantum register containing *n* qubits is naturally in a superposition of an exponential number of basis states (see Fig. 2). The aforementioned idea of R. Feynman of efficient quantum simulation is based on mapping of the exponentially large wave function of a studied system onto the exponentially large wave function of a quantum register.

**Fig. 2:**
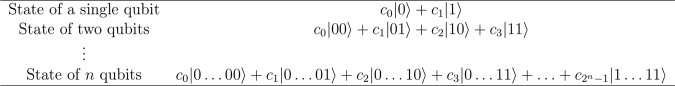
General states of one-, two-, and *n*-qubit quantum registers. The complex expansion coefficients *c*_*i*_ are supposed to satisfy the normalization.

In 2005, Aspuru-Guzik et al. presented in their seminal work the efficient (running in polynomial time and employing polynomial resources) quantum algorithm for FCI computations^5^, which is based on the phase estimation algorithm (PEA)^1^. Unlike newer variational quantum algorithms, which are designed for near-term noisy quantum devices, the quantum FCI algorithm will require fault-tolerant quantum computers and provides exact results^6^. It can be viewed in a simplified way as a time evolution of an initial guess of the exact state with subsequent inverse quantum Fourier transform^1^, switching from the time to the energy domain, followed by a measurement of a particular energy level, which causes a collapse of the initial state to the corresponding eigenstate. The quality of the initial guess state in fact determines the success probability of obtaining the desired energy level, which is proportional to |〈 *ψ*_init_ | *ψ*_exact_〉|^2^. An important point to stress is that the quantum FCI algorithm^5^ is efficient only as long as one is able to prepare a reasonably good initial guess state (in terms of the overlap with the exact eigenstate), which is also the problem studied by Takui et al.

Takui and colleagues^2^ employ and in several ways improve the adiabatic state preparation (ASP) method^5^. In the ASP method, one slowly varies the Hamiltonian of the quantum register, starting with a trivial one and the register in its (exactly known) ground state and ending with the final exact one, originally by means of the linear interpolation

1 $$H=(1-s){H}_{{{{{{\rm{init}}}}}}}+{{sH}}_{{{{{{\rm{exact}}}}}}}\,s:\,0\to 1.$$

If the change is slow enough (depending on the gap between the ground and the first excited state along the ASP path), the register remains in its ground state according to the adiabatic theorem^7^.

The authors of the aforementioned work^2^ numerically studied the performance of ASP on model strongly correlated molecules (N_2_, BeH_2_). Unlike weakly correlated systems, which have the dominant contribution to the wave function from the Hartree-Fock (mean-field) solution and which are routinely treated by classical approximate polynomially scaling methods^4^, these systems contain degenerate or quasidegenerate molecular orbitals and consequently several equally dominant configurations in a wave function expansion, which makes their electronic structure much more complex (see Fig. 3a). Moreover, many important chemical problems fall into this category, e.g., homolytic bond breaking/formation, open-shell and excited electronic states, transition-metal complexes, or transition states of chemical reactions. When the manifold of quasidegenerate orbitals is small (less than 20), it can be treated by FCI. However, when tens of orbitals are strongly correlated, the situation is much more complicated and here quantum algorithms are believed to become a game changer^6^.

**Fig. 3:**
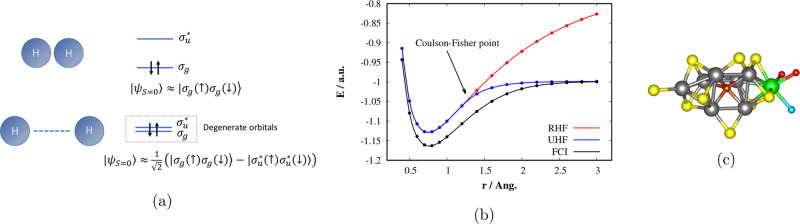
Complexity of the electronic structure of strongly correlated molecules. **a** The bond breaking process in H_2_ molecule as an example of the simplest strongly correlated problem, **b** comparison of restricted HF (RHF), unrestricted HF (UHF), and exact (FCI) ground state potential energy curves of H_2_ in cc-pVDZ basis, **c** Structure of the FeMoco cluster^9^, atom colors: nitrogen—blue, sulfur—yellow, oxygen—red, carbon—brown, iron—gray, molybdenum—green.

As an example of a strongly correlated molecular system, whose electronic structure was suggested as a prominent candidate for quantum algorithms by Reiher et al.^8^, we mention the Mo-dependent nitrogenase active site (Fe-Mo cofactor, FeMoco, see Fig. 3c). Enzyme nitrogenase is transforming N_2_ into two NH_3_ molecules under ambient conditions and understanding of this very complex process, whose mechanism is still not known, is of very high scientific and economic importance.

Let us demonstrate the main ideas of Takui et al.^2^ on the simplest strongly correlated problem, namely the bond breaking process in H_2_ molecule. It is well known that the restricted Hartree-Fock (RHF) method does not correctly describe this process^4^, since the symmetric doubly occupied bonding orbital (*σ*_*g*_) in the RHF single configuration causes an artificial non-zero contribution of the ionic form in the dissociation limit. On the other hand, the unrestricted Hartree-Fock (UHF) solution, in which *α* and *β* electrons are allowed to occupy different spatial orbitals describes the H_2_ homolytic bond breaking properly (see Fig. 3b). The UHF orbitals, which for the stretched bond region have the form of orbitals localized on individual atoms, break the spin symmetry and are called the broken-symmetry (BS) solution.

The authors of the aforementioned work^2^ suggest to use a single configuration made from localized BS singly occupied molecular orbitals as an initial state for ASP. Such states are equally simply preparable on quantum computers as HF configurations and clearly better describe the strongly correlated regions of potential energy surfaces. However, as mentioned above, they are not pure spin states. For this purpose, Takui et al.^2^ proposed a simple spin purification procedure based on an additional spin penalty term in the ASP Hamiltonian from Eq. 1, which does not bring large computational overhead.

As is numerically proven in their work, ASP with BS initial states and spin penalty terms outperforms ASP with RHF initial states in strongly correlated regimes, which would correspond to bond lengths longer than for the Coulson-Fisher point in case of our simple H_2_ example (see Fig. 3b). On the other hand, the later outperforms the former in weakly correlated regimes (shorter-than-Coulson-Fisher bonds). Takui et al.^2^ therefore proposed to use the diradical character measure based on the UHF natural orbital occupation numbers as a simple heuristic, which can switch between the ASP procedure with RHF or BS initial states. Another important point to stress is that the authors in their work^2^ rely only on cheap preliminary Hartree-Fock calculations performed on a classical computer. The rest would be performed on a quantum computer. In this study, its action was simulated numerically. Since the total ASP time *T*_tot_ depends on the minimum energy gap between the ground and the first excited state, the authors provide a practical guess for *T*_tot_, which is based on the HOMO–LUMO gaps and renders states with large-enough overlaps with the exact wave functions. Last but not least, the authors numerically studied the effect of different non-linear ASP interpolation schemes and provide optimal ones for ASP with RHF as well as BS initial states.

In summary, Takui et al. in their work^2^ numerically studied the ASP procedure of correlated electronic wave functions and provide several concrete computational conditions. Despite the fact that these conditions are optimal for the studied models of strongly correlated problems and their portability to other more complex molecules must be probed by further theoretical and experimental studies, the work represents an important step towards practical applications of quantum computing in chemistry. The disadvantage of ASP in combination with PEA is that it requires deep circuits and consequently fault-tolerant quantum computers. It would thus be interesting to see, if the ideas of variational quantum algorithms^6^ might be combined with ASP to decrease the number of quantum gates and allow for simulations on near-term noisy quantum devices.
